# Characterization and potential role of microRNA in the Chinese dominant malaria mosquito *Anopheles sinensis* (Diptera: Culicidae) throughout four different life stages

**DOI:** 10.1186/s13578-018-0227-1

**Published:** 2018-04-12

**Authors:** Xinyu Feng, Jiatong Wu, Shuisen Zhou, Jingwen Wang, Wei Hu

**Affiliations:** 1 National Institute of Parasitic Diseases, Chinese Center for Disease Control and Prevention, Key Laboratory of Parasite and Vector Biology, National Health and Family Planning Commission, WHO Collaborating Center for Tropical Diseases, National Center for International Research On Tropical Diseases, Shanghai, 200025 People’s Republic of China; 2 Joint Research Laboratory of Genetics and Ecology on Parasites-hosts Interaction, National Institute of Parasitic Diseases-Fudan University, Shanghai, 200025 People’s Republic of China; 3 State Key Laboratory of Genetic Engineering, Ministry of Education Key Laboratory of Contemporary Anthropology, Collaborative Innovation Center for Genetics and Development, School of Life Sciences, Fudan University, Shanghai, 200438 People’s Republic of China; 4 Department of Microbiology and Microbial Engineering, School of Life Science, Fudan University, Shanghai, 200433 China

**Keywords:** microRNA, Non-coding RNA, *Anopheles sinensis*, Stage-specific, Development, Malaria, China

## Abstract

**Background:**

microRNAs (miRNAs) are one kind of small non-coding RNAs widely distributed in insects. Many studies have shown that miRNAs play critical roles in development, differentiation, apoptosis, and innate immunity. However, there are a few reports describing miRNAs in *Anopheles sinensis*, the most common, and one of the dominant malaria mosquito in China. Here, we investigated the global miRNA expression profile across four different developmental stages including embryo, larval, pupal, and adult stages using Illumina Hiseq 2500 sequencing.

**Results:**

In total, 164 miRNAs were obtained out of 107.46 million raw sequencing reads. 99 of them identified as known miRNAs, and the remaining 65 miRNAs were considered as novel. By analyzing the read counts of miRNAs in all developmental stages, 95 miRNAs showed stage-specific expression (q < 0.01 and |log2 (fold change)| > 1) in consecutive stages, indicating that these miRNAs may be involved in critical physiological activity during development. Sixteen miRNAs were identified to be commonly dysregulated throughout four developmental stages. Many miRNAs showed stage-specific expression, such as asi-miR-2943 was exclusively expressed in the embryo stage, and asi-miR-1891 could not be detected in larval stage. The expression of six selected differentially expressed miRNAs identified by qRT-PCR were consistent with our sequencing results. Furthermore, 5296 and 1902 target genes were identified for the dysregulated 68 known and 27 novel miRNAs respectively by combining miRanda and RNAhybrid prediction. GO annotation and KEGG pathway analysis for the predicted genes of dysregulated miRNAs revealed that they might be involved in a broad range of biological processes related with the development, such as membrane, organic substance transport and several key pathways including protein processing in endoplasmic reticulum, propanoate metabolism and folate biosynthesis. Thirty-two key miRNAs were identified by microRNA-gene network analysis.

**Conclusion:**

The present study represents the first global characterization of *An. sinensis* miRNAs in its four developmental stages. The presence and differential expression of *An. sinensis* miRNAs imply that such miRNAs may play critical roles in *An. sinensis* life cycle. A better understanding of the functions of these miRNAs will have great implication for the effective control of vector population and therefore interrupting malaria transmission.

**Electronic supplementary material:**

The online version of this article (10.1186/s13578-018-0227-1) contains supplementary material, which is available to authorized users.

## Background

*Anopheles sinensis* is one of the most important disease vectors in China. It is considered as the primary vector of *P. vivax* malaria due to its wide distribution and high density in central China where several malaria outbreaks occurred in history [1, 2]. Like many mosquitoes, *An. sinensis* goes through four life stages that include egg, larva, pupa and the adult. During these developmental periods, a fine-tuning complex biological process, such as embryogenesis, organ differentiation, and metamorphosis, etc., is completed.

For the past decades, facilitated by high-throughput sequencing technology, a large number of small non-coding RNAs (ncRNAs) have been discovered in many species [3, 4]. Amongst them, microRNAs (miRNAs) have become the most popular research topic for its critical role in regulating important biological events at the post-transcriptional level. Mature miRNAs are single-stranded, evolutionarily conserved endogenous small ncRNAs of approximately 22 nucleotides (nt) in length, which could lead to transcriptional decay through translational inhibition, transcript degradation or both [5, 6].

The first miRNA repertoire of mosquito species was reported from the African malaria vector *Anopheles gambiae* [7]. Since then, mosquito miRNAs have been identified and characterized in a number of mosquito species, including *Anopheles stephensi* [8, 9], *Aedes aegypti* [10–12], *Culex fatigans* [13–15], and many others, but not yet in *An. sinensis*. Several studies suggested that miRNAs may contribute to an important regulatory role in the post-transcriptional level in developmental stages. For instance, temporal and stage specific expression of miRNAs has been identified in the embryo, larval, pupal and adult stage of *Ae. albopictus* [16], *An. anthropophagus* [17], and *An. stephensi* [9]. Among them, the expression of aal-miR-286b was increased in the embryo, while aal-miR-2942 had the highest level of expression in larvae although it was normally expressed at the embryo, pupal and adult stages [16]. In addition, ast-miR-1891, ast-miR-190-3p, ast-miR-285, ast-miR-988-3p, and ast-miR-989 were absent in the larval stage, while ast-miR-8-3p was the most abundant in both male and female larval stage [9]. These results indicated that dysregulated miRNAs might play pivotal roles in embryogenesis and metamorphosis throughout development. However, the exact functions of these miRNAs remain to be determined.

In this study, we provided a comprehensive repertoire of *An. sinensis* miRNAs and investigated the differential expression of miRNAs using Solexa sequencing technology, with an aim to identify key regulatory miRNAs through development stages. Selected candidate miRNAs were then validated by qRT-PCR in samples that have been used in sequencing. To explore the potential gene regulatory networks, 32 dysregulated miRNAs were defined as key miRNAs during development and then subjected to miRNA-gene network analysis in terms of developmental pathway analysis. The data described here served as an important step towards the understanding of gene expression modulation by miRNAs in *An. sinensis*, which will further advance functional studies for novel mosquito control approaches developing and promote elimination campaign in China.

## Methods

### Mosquito rearing and sample preparation

*Anopheles sinensis* (China strain) from insectary were reared at 28 ± 2 °C, 70–75% humidity in the Vector Control Reference Laboratory at the National Institute of Parasitic Diseases (NIPD), Shanghai, China. Adult mosquitoes were maintained in screened cages, and provided constant access to water and glucose-soaked sponges.

Sample collections from different developmental stages of *An. sinensis* for high throughput sequencing are briefly described below. Embryos were collected within 24 h after placing a damp collection filter paper within the cage and were pooled to represent the embryonic stage. In the larval sample collection, we used mixed larval sample (I–IV instars) including early and late larval samples. Pupal samples were collected from a pool of varied ages. Female adults were collected after emerging one to 3 days. All samples were flash frozen in liquid nitrogen immediately following collection, and then stored at − 80 °C until for RNA isolation.

### RNA extraction and small RNA sequencing

Briefly, total RNAs were extracted separately from the four different life cycle stages of *An. sinensis* (eggs, larvae, pupae and unfed adult females) using TRIzol reagent (Invitrogen, USA) according to the manufacturer’s protocol. RNA purity was checked using the NanoPhotometer^®^ spectrophotometer (IMPLEN, CA, USA). RNA degradation and contamination were monitored on 1% agarose gels. RNA concentration was measured using Qubit^®^ RNA Assay Kit in Qubit^®^ 2.0 Fluorometer (Life Technologies, CA, USA). RNA quality assessment was determined by using Agilent 2100 Bioanalyzer RNA Nano 6000 kit of the Agilent Bioanalyzer 2100 system (Agilent Technologies, CA, USA). No rRNA depletion was performed. RNA extracts of the four life stages were sent to Newgenes Inc (Shanghai, China) for small RNA sequencing. A total amount of 3 µg of total RNA was ligated with 3′ and 5′ adaptors followed by reverse transcription using RT primers. The libraries were size-selected for sequencing of RNA fragments of 18–30 nucleotides. These small RNA libraries were then sequenced using Illumina Hiseq 2500 platform (Illumina Inc., USA).

### Small RNA sequencing data processing and analysis

Following sequencing, raw data (raw reads) of fastq format were firstly processed through custom perl and python scripts. In this step, clean data (clean reads) were obtained by removing reads containing ploy-N, with 5′ adapter contaminants, without 3′ adapter or the insert tag, containing ploy A or T or G or C and low quality reads from raw data. At the same time, Q20, Q30, and GC-content of the raw data were calculated. Then, a certain range of clean reads from each stage was combined to do all the downstream analyses. All reads were mapped to the *An. sinensis* strain China genome [GenBank: GCA_000441895.2] by Bowtie [18] without mismatch to analyze their expression and distribution on the reference.

For identification of known and novel miRNA sequences present in the four life stage datasets. Firstly, after removing tags originating from protein-coding genes, repeat sequences, rRNA, tRNA, snRNA, and snoRNA, small RNA tags were mapped to RepeatMasker, Rfam database or those types of data from *Anopheles gambiae*. Mapped small RNA tags were used to look for known miRNA using miRBase21 [19] as reference. Custom scripts adapted from quantitative script of miRDeep2 were used to obtain the miRNA counts as well as base bias on the first position of identified miRNAs with certain length and on each position of all identified miRNAs. The reads that did not yield a match were used to predict novel miRNAs. The available software miREvo [20] and mirdeep2 [21] were integrated to predict novel miRNA from the small RNA tags unannotated in the former steps. A number of stringent criterions were used for evaluating whether novel miRNA candidates were genuine miRNAs, such as exploring the secondary structure by RNAfold, calculating minimum free energy (MFE) and avoiding isolated base pairs. In each library, same sequences were pooled together to generate expression files (fasta) with their read count and unique ID used for further analysis.

All identified known miRNAs were named according to their most similar miRNAs in the miRNA database (miRBase) release 21 (June 2017). All identified novel miRNAs were named sequentially following the asi-mir-nov1. Raw sequencing data were prepared to submit to the National Center for Biotechnology Information (NCBI).

### Quantification of miRNA and differential expression analysis

In order to analyze the relative abundance and expression level of miRNAs, tags per million of total RNA reads (TPM) for each miRNA in all four libraries were calculated. TPM was compared between the libraries to identify miRNAs differentially expressed between two consecutive life stages in the mosquito. Differential expression analysis of two samples was performed using the DEGseq [22] R package. p value was adjusted using q value [23]. q < 0.01 and |log2 (fold change)| > 1 was set as the threshold for significantly differential expression by default.

### miRNA target prediction, GO and KEGG enrichment analysis

To predict the potential targeted genes of miRNA, the targeted mRNAs of differentially expressed known and novel miRNAs were predicted by combining miRanda [24] and RNAhybrid [25] to select and assess overlapping predicted targets between each program. Briefly, 3′UTR of *An. gambiae* downloaded from vectorbase were used for the prediction. The targets showing complementarity with miRNA seed region and binding energy ≤ 20 kcal/mol were selected. The predicted targets were used for further GO and KEGG enrichment analysis.

Gene ontology (GO) enrichment analysis was used on the target gene candidates of differentially expressed miRNAs (“target gene candidates” in the following). GOseq based Wallenius non-central hyper-geometric distribution [26], which could adjust for gene length bias, was implemented for GO enrichment analysis. GO terms with corrected p value less than 0.05 were considered significantly enriched by target genes. KEGG pathway analyses were performed using the Kyoto Encyclopedia of Genes and Genomes (KEGG) (http://www.genome.ad.jp/kegg/) database [27]. In this study, we used KOBAS [28] software to test the statistical enrichment of the target gene candidates in KEGG pathways, with the threshold of corrected p < 0.05.

### miRNA-gene network analysis

To identify the key miRNAs in *An. sinensis* development, microRNA-gene network analysis was conducted for the commonly dysregulated miRNAs identified from the microRNA expression files, the exclusively expressed miRNAs in certain stages (TPM > 10) and the most significantly over/under-expressed miRNAs (>tenfold) and their predicted target developmental genes. Developmental genes of *An. gambiae* were accessed based on gene annotation in VectorBase [29] and through intensive literature surveys [30–33]. Interactive networks between key miRNAs and their target developmental genes were constructed by using Cytoscape [34].

### miRNA expression validated by quantitative RT-PCR

To validate the presence and expression level of the identified miRNAs, nine miRNAs with differential expression were selected for qRT-PCR as described previously [35]. The total RNA was extracted using TRIzol Reagent (Invitrogen, USA). The reverse transcription reaction was performed with RevertAid First Strand cDNA Synthesis Kit (Fermentas, USA), and the reverse-transcribed products were used as the template for qRT-PCR with miRNA-specific primers (Additional file 1: Table S1). All reactions were assayed in three biological and technical replications, and performed in an ABI-7300 (Applied Biosystems, USA) using SYBR Green qPCR kit (Thermo, USA). Quantitative measurements were normalized to the internal control of *U6* small nuclear (*U6*), and relative expression was calculated by using the 2^−ΔΔCt^ method [14].

## Results

### Small RNAs sequence of the four libraries

We performed small RNA sequencing from eggs, larva, pupa and adult mosquito batches to identify *An. sinensis* miRNAs expressed during developmental stages. All sequencing data can be found in NCBISRA (PRJNA412443). In total, sequencing of four libraries yielded about 10.75 × 10^7^ reads. After trimming the adaptors, 1.99 × 10^7^ (96.71%), 2.85 × 10^7^ (92.81%), 2.91 × 10^7^ (86.06%) and 2.06 × 10^7^ (92.38%) clean reads from ASE (egg), ASL (larva), ASP (pupa) and ASA (adult) libraries respectively were utilized for further processing (Table 1). The total number of the reads mapped to *An. sinensis* genome constitutes 82.53, 77.61, 80.55 and 85.75% of the total high-quality sRNA (small RNA) reads from eggs, larvae, pupae, and adult libraries, respectively (Table 1).

**Table 1:** Summary of small RNA sequencing data analysis for the four life stage libraries of *An. sinensis*

Sample	Total reads	Clean reads	Total sRNA	Mapped reads to the genome (%)
ASE	20,631,008	19,952,620	17,463,602	13,553,741 (77.61%)
ASL	30,729,308	28,519,352	17,429,555	14,384,064 (82.53%)
ASP	33,772,307	29,064,021	16,165,081	13,860,873 (85.75%)
ASA	22,325,688	20,624,870	14,026,979	11,299,314 (80.55%)

ASE: *An. sinensis* egg, ASL: *An. sinensis* lave, ASP: *An. sinensis* pupa, ASA: *An. sinensis* female adult

### Length distribution of small non-coding RNAs in the four libraries

**Fig. 1:**
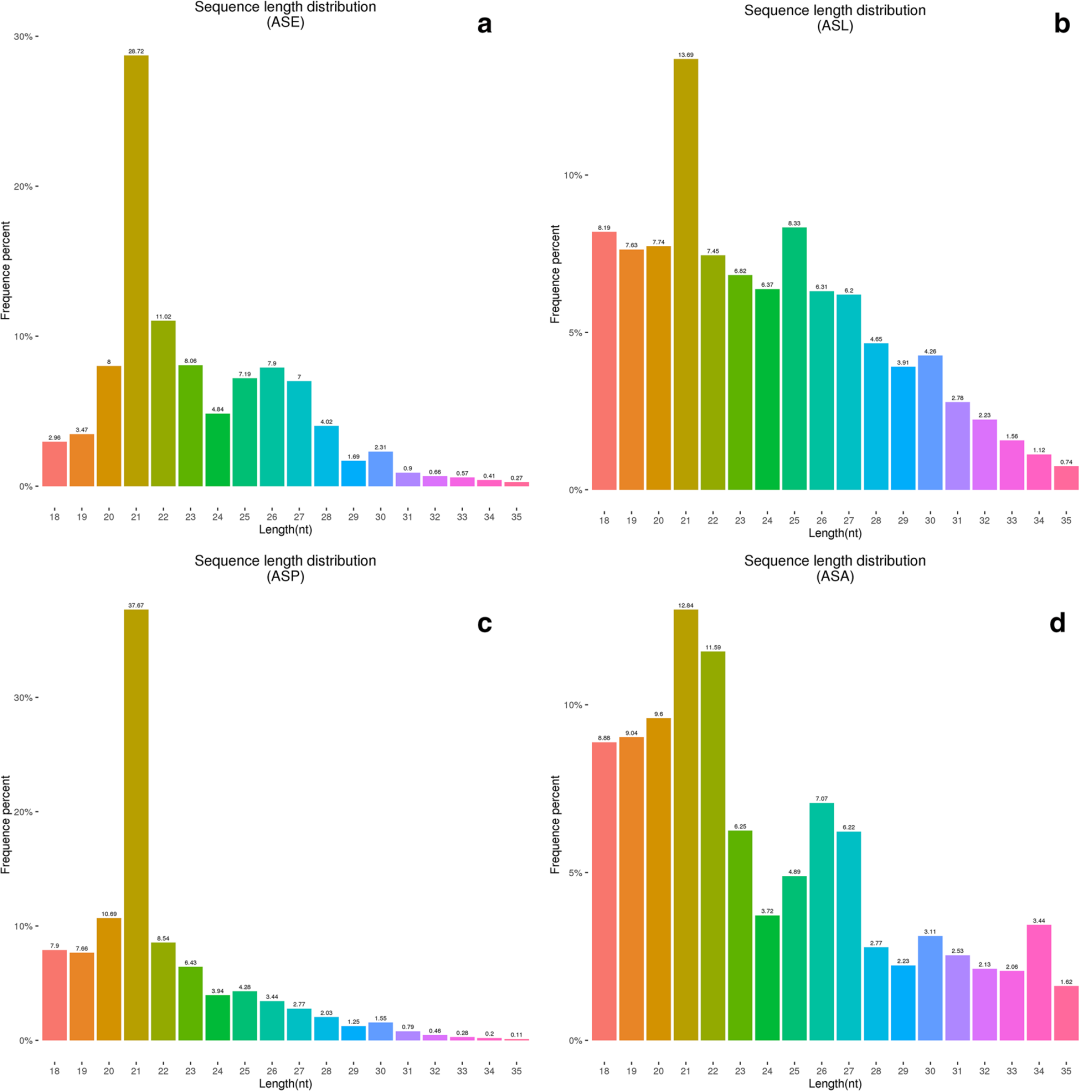
Length distribution of small RNA reads in **a** *An. sinensis* embryo library (ASE), **b** *An. sinensis* larval library (ASL), **c** *An. sinensis* pupal library (ASP), **d** *An. sinensis* female adult library (ASD). X axis represents small RNA read lengths in base pairs while Y axis represents number of reads

Among the libraries, size distribution of reads varied between 18 and 35 nt. We found that one peak with 21 nt in all libraries, followed by another peak with 20–23 nt which represents miRNAs (Fig. 1). This was consistent with the typical size of miRNAs in other reports. There was also a peak at 24–27 nt in almost all developmental libraries, which corresponds to piRNA-like small RNAs suggesting a possible role in embryogenesis and maintaining genome stability during development.

### Annotation of small non-coding RNAs in the four libraries

**Fig. 2:**
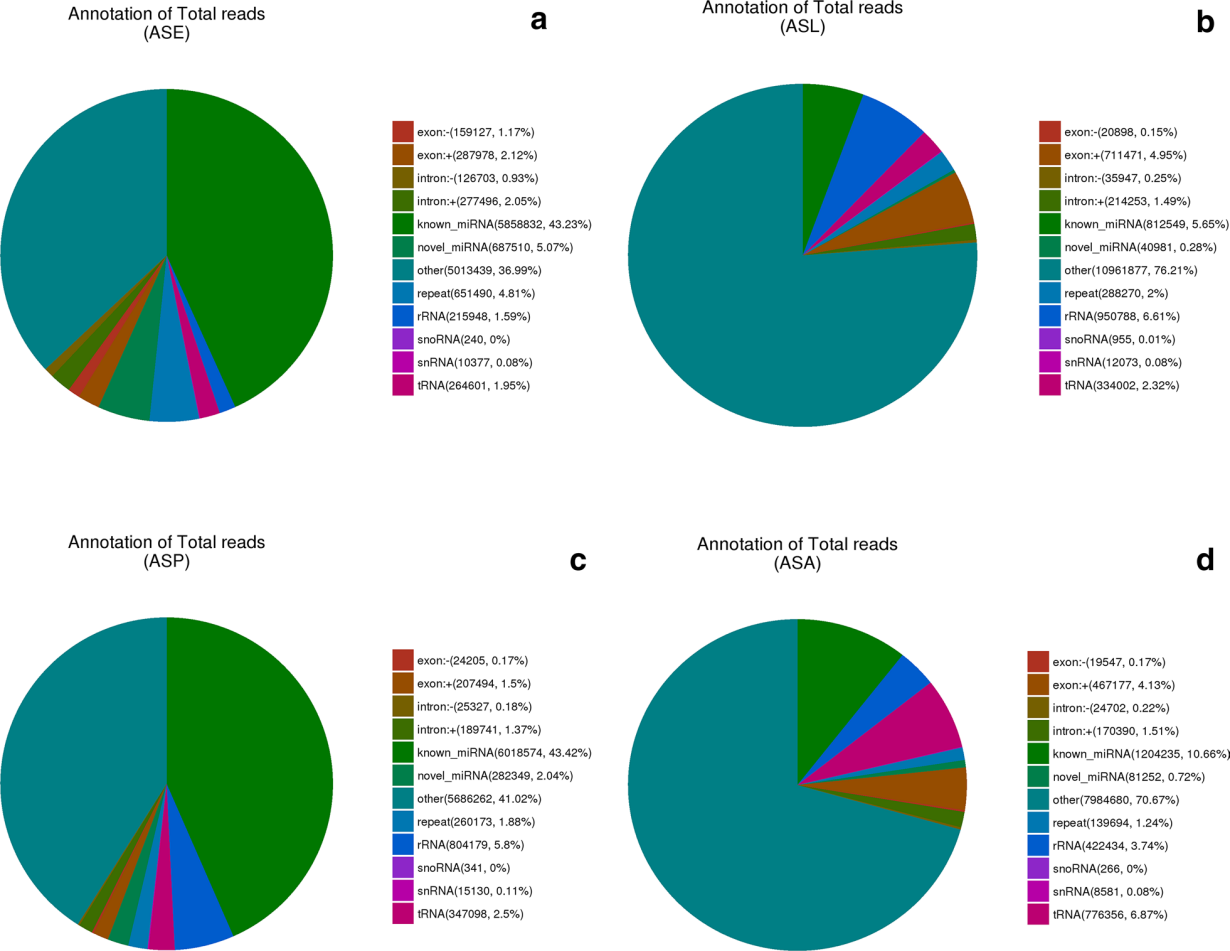
Annotation of small non-coding RNAs from the four life stage libraries of *An. sinensis*. **a** *An. sinensis* embryo library (ASE), **b** *An. sinensis* lave library (ASL), **c** *An. sinensis* pupa library (ASP). **d** *An. sinensis* adult library (ASA). The total reads can be divided into 12 categories; exon−, exon+, intron−, intron+, known miRNA, novel miRNA, others, repeat, rRNA, snoRNA, snRNA and tRNA. The others referred to the other class of small non-coding RNAs and the reads not aligned to RNA families’ database

In order to annotate other small non-coding RNAs in the four libraries, all clean reads were aligned against Rfam version 11 [36]. As showed in Fig. 2, the most abundant class of small non-coding RNAs in the eggs’ library was miRNAs (50.10%). However, rRNAs were most abundant in the larvae (6.61%) and pupae library (5.8%), and tRNAs in the adult females’ library (6.87%).

### Identification of miRNAs in the four life stages libraries of *An. sinensis*

Based on previously known mosquito mature miRNAs in miRBase release 21 (June 2017), we discovered 99 known miRNAs in *An. sinensis* (Table 2) throughout four development stages. Reads that do not match any known miRNA sequences were further employed by miREvo and mirdeep2 to investigate the folding properties of precursors or hairpins for novel miRNAs. Finally, 65 mature miRNAs were identified as novel (default parameters of miREvo and mirdeep2) (Additional file 2: Table S2). Thus, a total of 164 *An. sinensis* miRNAs were identified in four life stages libraries of *An. sinensis.*

**Table 2:** Known miRNAs identified in four life stages libraries of *An. sinensis*

miRNA name	Sequences	Length	Location	Tags per million (TPM)			
				ASE	ASL	ASP	ASA
asi-miR-276-3p	UAGGAACUUCAUACCGUGCUC	21	KE525297.1:1458670..1458650:+	167,805.76	318,655.14	451,492.06	316,672.78
asi-miR-9a	UCUUUGGUUAUCUAGCUGUAUGA	23	KE525001.1:301001..301023:−	141,229.57	137,568.26	57,223.48	27,174.76
asi-miR-1	UGGAAUGUAAAGAAGUAUGGAG	22	KE524975.1:1035748..1035769:−	187,484.83	88,731.09	100,424.03	115,685.16
asi-miR-100	AACCCGUAGAUCCGAACUUGUG	22	KE525346.1:1178073..1178052+	5057.84	38,962.34	97,988.23	78,039.03
asi-miR-8-3p	UAAUACUGUCAGGUAAAGAUGUC	23	KE525347.1:277471..277449:+	2933.19	35,223.24	16,842.53	56,364.03
asi-miR-999	UGUUAACUGUAAGACUGUGUCU	22	KE525161.1:40638..40659:-	17,377.27	34,087.38	38,041.81	55,432.34
asi-miR-11-3p	CAUCACAGUCUGAGUUCUUGCU	22	KE525297.1:1145644..1145665−	21,936.31	33,023.90	11,854.78	18,914.99
asi-miR-184	UGGACGGAGAACUGAUAAGGGC	22	KE525421.1:213400..213421:−	27,788.31	26,840.31	15,801.40	19,820.19
asi-miR-7	UGGAAGACUAGUGAUUUUGUUGU	23	KE525340.1:256575..256553:+	69,849.63	25,689.28	18,252.89	35,200.06
asi-miR-281-3p	UGUCAUGGAAUUGCUCUCUUUA	22	KE525349.1:2646148..2646169:−	5649.35	25,360.08	6667.99	34,188.91
asi-miR-9c-5p	UCUUUGGUAUUCUAGCUGUAGA	22	KE525317.1:660185..660164:+	25,959.34	23,472.43	13,744.82	18,872.14
asi-miR-263a-5p	AAUGGCACUGGAAGAAUUCACGG	23	KE525098.1:220160..220138:+	53,603.64	19,911.93	33,053.10	12,860.57
asi-miR-306	UCAGGUACUGGAUGACUCUCAG	22	KE525317.1:662779..662758:+	26,686.89	17,783.81	6780.18	12,437.57
asi-miR-305-5p	AUUGUACUUCAUCAGGUGCUCUGG	24	KE524841.1:15363..15386:−	4162.66	17,523.48	19,993.02	11,341.51
asi-miR-9b	ACUUUGGUGAUUUUAGCUGUAUG	23	KE525317.1:663617..663595:+	13,191.15	13,483.19	3319.73	10,659.89
asi-miR-10	ACCCUGUAGAUCCGAAUUUGUU	22	KE525233.1:711575..711554:+	75,624.85	12,681.21	8573.87	8382.08
asi-miR-1174	UCAGAUCUACUUCAUACCCAUG	22	KE525248.1:186355..186334+	5123.51	11,635.24	1359.33	7155.15
asi-miR-279	UGACUAGAUCCACACUCAUUAA	22	KE525236.1:156609..156588+	7462.21	10,187.69	8815.69	7220.59
asi-miR-281-5p	AAGAGAGCUAUCCGUCGAC	19	KE525349.1:2646188..2646206:−	1793.94	8863.89	2189.07	10,265.71
asi-miR-278-3p	UCGGUGGGACUUUCGUCCGUUU	22	KE525347.1:4349292..4349271:+	579.48	8851.05	2287.00	2259.11
asi-miR-13-3p	UAUCACAGCCAUUUUGACGAGUU	23	KE525350.1:1783388..1783366+	9781.06	7638.14	4976.66	4480.83
asi-miR-275-3p	UCAGGUACCUGAAGUAGCGC	20	KE524841.1:27273..27292:−	511.15	6568.82	5975.96	5463.93
asi-let-7	UGAGGUAGUUGGUUGUAUAGU	21	KE525346.1:11781486..1181466:+	61.76	6319.01	12,631.90	15,097.09
asi-miR-1175-5p	AAGUGGAGUAGUGGUCUCAUCG	22	KE525248.1:186488..1864678:+	1350.50	5971.13	98.09	1610.20
asi-miR-2765	UGGUAACUCCACCACCGUUGGC	22	KE524732.1:275289..27549:−	35.18	5915.09	37.08	14.02
asi-miR-2c	UAUCACAGCCAGCUUUGAUGAGC	23	KE525350.1:1783822..1783800+	5517.23	5218.17	4288.76	2824.67
asi-miR-2b	UAUCACAGCCAGCUUUGAUGAGCU	24	KE525350.1:1783823..1783800+	5510.66	5214.67	4290.50	2825.45
asi-miR-2a-3p	UAUCACAGCCAGCUUUGAAGAGC	23	KE525350.1:1783565:1783543:+	3337.86	4938.00	2844.96	2808.31
asi-miR-14	UCAGUCUUUUUCUCUCUCCUAU	22	KE525231.1:338814..338835:−	4248.19	4844.61	9771.57	11,007.32
asi-miR-34-5p	UGGCAGUGUGGUUAGCUGGUUG	22	KL645799.1:184..163:+	35.18	4643.82	102.85	16,234.44
asi-miR-375	UUUGUUCGUUUGGCUCGAGUUA	22	KE525304.1.363119..363140:−	2327.29	2722.32	7.45	25.71
asi-miR-8-5p	CAUCUUACCGGGCAGCAUUAGA	22	KE525347.1:277430..277409:+	1309.69	2236.69	1534.12	4757.38
asi-miR-1175-3p	UGAGAUUCUACUUCUCCGACUUAA	24	KE525248.1:186528..186505:+	50.35	2225.02	88.11	925.46
asi-miR-315-5p	UUUUGAUUGUUGCUCAGAAAGC	22	KE524190.1:262690..262711:−	12,578.06	2041.74	778.55	765.76
asi-miR-252-5p	UAAGUACUAGUGCCGCAGGAG	21	KE525267.1:347072..347092:−	2708.81	1847.96	1507.97	2164.85
asi-miR-317	UGAACACAUCUGGUGGUAUCUCAGU	25	KE524744.1:7138..7162:−	7.04	1746.39	127.72	2322.21
asi-miR-190	AGAUAUGUUUGAUAUUCUUGGUUG	24	KE525352.1:3462214..3462237:−	2311.34	1737.06	1090.57	2010.61
asi-miR-283	UAAAUAUCAGCUGGUAAUUCU	21	KE524901.1:23378..23398:−	2555.73	1557.28	621.19	1756.65
asi-miR-957	UGAAACCGUCCAAAACUGAGGC	22	KE525321.1:54139..54160:−	237.51	1363.50	1523.98	1921.02
asi-miR-998	UAGCACCAUGAGAUUCAGC	19	KE524842.1:1145486..1145504:+	621.23	1236.25	352.11	509.47
asi-miR-263b-5p	CUUGGCACUGGGAGAAUUCACAG	23	KE524589.1:97550..97528:+	3141.31	1188.39	2647.35	1553.33
asi-miR-71-5p	AGAAAGACAUGGGUAGUGAGAU	22	KE525350.1:1781736..1781715:+	220.47	968.92	375.73	419.88
asi-miR-92b-3p	AAUUGCACUUGUCCCGGCCUGC	22	KE523980.1:64015..64036:+	647.34	938.57	175.74	151.91
asi-miR-277-3p	UAAAUGCACUAUCUGGUACGAC	22	KE524932.1:25145..25166:–	891.26	797.32	15455.79	23,261.05
asi-miR-71-3p	UCUCACUACCUUGUCUUUCAUG	22	KE525350.1:1781809..1781788:+	346.19	652.56	309.17	257.85
asi-miR-125-5p	UCCCUGAGACCCUAACUUGUGA	22	KE525346.1:1182133..1182112:+	9.69	644.39	1732.05	1625.78
asi-miR-970	UCAUAAGACACACGCGGCUAU	21	KE525003.1:917073..917053:+	603.56	630.38	650.98	843.66
asi-miR-981	UUCGUUGUCGACGAAACCUGCA	22	KE524855.1:1042040..1042019:+	686.74	611.71	1152.22	895.08
asi-miR-31	UGGCAAGAUGUUGGCAUAGCUGA	23	KE524855.1:565934..565957:−	178.72	584.86	403.62	531.28
asi-miR-iab-4-5p	ACGUAUACUGAAUGUAUCCUGA	22	KE525233.1:295585..295606:−	3101.28	573.18	135.96	222.80
asi-miR-12-5p	UGAGUAUUACAUCAGGUACUGGU	23	KE524901.1:21292..21314:+	512.40	554.50	502.18	771.99
asi-miR-92a-3p	UAUUGCACUUGUCCCGGCCUA	21	KL646680.1:1643..1623:+	572.28	552.17	152.45	129.31
asi-miR-9c-3p	UAAAGCUUUAGUACCAGAGGUC	22	KE525317.1:660231..660210:+	383.40	551.00	248.64	310.82
asi-miR-133	UUGGUCCCCUUCAACCAGCUGU	22	KE524620.1:429840..429861:−	264.41	441.27	500.12	662.93
asi-miR-305-3p	CGGCACAUGUUGGAGUACACUUA	23	KE524841.1:15325..15347:−	160.90	429.59	460.98	166.71
asi-miR-282-5p	AAUCUAGCCUCUCCUAGGCUUUGUCUG	27	KE525264.1:323815..323789:+	1.25	391.07	73.37	52.19
asi-miR-996	UGACUAGAUUACAUGCUCGUC	21	KE525286.1:157381..157361:+	107.26	347.88	231.05	482.98
asi-miR-87	GGUGAGCAAAUAUUCAGGUGU	21	KE524974.1:1235367..1235347:+	320.39	326.87	406.47	736.16
asi-miR-993	UACCCUGUAGUUCCGGGCUUUU	22	KE525233.1:748304..748325:−	3470.14	261.49	182.08	255.51
asi-miR-989	UGUGAUGUGACGUAGUGGUAC	21	KE525091.1:234485..234465:+	420.77	246.32	956.98	20,202.68
asi-miR-1890	UGAAAUCUUUGAUUAGGUCUGG	22	KE524970.1:186795..186816:+	1025.26	245.15	900.89	499.34
asi-miR-137	UAUUGCUUGAGAAUACACGUAG	22	KE525275.1:771433..771454:−	745.69	222.97	334.05	453.38
asi-miR-932-5p	UCAAUUCCGUAGUGCAUUGCAG	22	KE525415.1:21588..321609:−	22.67	221.80	552.42	627.10
asi-miR-965	UAAGCGUAUAGCUUUUCCCAUU	22	KE524970.1:286640..286719:+	137.60	171.60	72.74	103.61
asi-miR-276-5p	AGCGAGGUAUAGAGUUCCUAC	21	KE525297.1:1458630..1458610:+	41.75	128.41	118.69	180.73
asi-miR-1000	AUAUUGUCCUGUCACAGCAGU	21	KE524842.1:961875..961895:+	912.06	126.08	133.27	148.01
asi-miR-13a-3p	UAUCACAGCCAUUUUGAUGAGU	22	KE525350.1:1782958..1782938:+	33.62	74.71	41.04	49.86
asi-miR-2944a-5p	GAAGGAACUUCUGCUGUGAUCUGA	24	KE525343.1:1543524..1543501:+	42,259.28	64.21	17.75	61.54
asi-miR-285	UAGCACCAUUCGAAAUCAGUAC	22	KE525331.1:1654228..1654249:+	7.97	52.53	617.71	701.10
asi-miR-79-3p	UAAAGCUAGAUUACCAAAGCAU	22	KE525317.1:663064..663043:+	65.52	51.36	30.90	22.59
asi-miR-210	CUUGUGCGUGUGACAACGG	19	KE525157.1:631346..631328:+	39.25	49.03	119.48	330.30
asi-miR-124	UAAGGCACGCGGUGAAUGC	19	KE525231.1:190116..190134:−	91.16	47.86	43.58	50.64
asi-miR-988-5p	GUGUGCUUUGUGACAAUGAGA	21	KE525307.1:126115..126135:+	75.21	40.86	36.61	70.11
asi-miR-307	CACAACCUCCUUGAGUGAGCGA	22	KE525347.1:4066618..4066639:−	11.88	29.18	32.64	41.29
asi-miR-252-3p	CUGCUGCCCAAGUGCUUAUCG	21	KE525267.1:347039..347059:−	6.25	22.18	10.30	11.69
asi-miR-988-3p	CCCCUUGUUGCAAACCUCACGC	22	KE525307.1:126074..106095:+	17.98	18.68	12.36	24.15
asi-miR-33	GUGCAUUGUAGUUGCAUUGCA	21	KE524855.1:1060739..1060719:−	0.47	8.17	1.43	2.34
asi-miR-2944b-5p	GAAGGAACUCCCGGUGUGAUAUA	23	KE525324.1:712584..712563+	2128.40	5.84	2.38	7.01
asi-miR-79-5p	GCUUUGGCGCUUUAGCUGUAUGA	23	KE525317.1:663023..663001:+	16.89	5.84	1.43	6.23
asi-miR-929	AUUGACUCUAGUAGGGAGUCC	21	KE524723.1:863..843:+	11.26	5.84	11.09	12.46
asi-miR-277-5p	CGUGUCAGAAGUGCAUUUACA	21	KE524932.1:25192..25212:−	4.53	4.67	97.93	52.97
asi-miR-286b	UGACUAGACCGAACACUCGUAUCCC	25	KE525324.1:712419..712396:+	10,890.76	2.33	10.30	24.93
asi-miR-2944b-3p	UAUCACAGCAGUAGUUACCUGA	22	KE525324.1:712629..712610	124.93	2.33	0.16	0.78
asi-miR-193	UACUGGCCUACUAAGUCCCAAC	22	KE524784.1:191638..191659:−	39.72	2.33	251.96	24.15
asi-miR-219	UGAUUGUCCAAACGCAAUUCUUG	23	KE525339.1:431460..431438:+	2.50	2.33	5.23	0.78
asi-miR-286a	UGACUAGACCGAACACUCGCGUCCU	25	KE525343.1:1543040..1543016:+	227.04	1.17	0.32	0.00
asi-miR-263b-3p	UGGAUCUUUUCGUGCCAUCGU	21	KE524589.1:97592..97572:+	9.54	1.17	3.01	1.56
asi-miR-308-3p	AAUCACAGGAGUAUACUG	18	KE524853.1:228639..228622:+	2.19	1.17	1.27	3.12
asi-miR-4968-3p	CAGCAACAGCAGCAGCAGCAGA	22	KE525077.1:54358:54337:+	0.31	1.17	0.00	0.00
asi-miR-275-5p	CGCGCUAAGCAGGAACCGAGAC	22	KE524841.1:27320..27337:−	0.00	1.17	0.63	0.00
asi-miR-2943	UUAAGUAGGCACUUGCAGGCAAA	23	KE525350.1:266997..266975+	5825.57	0.00	0.00	0.00
asi-miR-2944a-3p	UAUCACAGUAGUUGUACUUUAA	22	KE525324.1:712814..712793:+	225.32	0.00	0.16	0.78
asi-miR-309a	UCACUGGGCAAAGUUUGUCGC	21	KE525324.1:713000..712980:+	65.67	0.00	0.00	3.90
asi-miR-315-3p	CUUUCGAGCAGUAAUCAAAGUC	22	KE524190.1:262648..262669:−	2.19	0.00	0.00	0.78
asi-miR-1891	UGAGGAGUUAAUUUGCGUGUUU	22	KE525346.1:7507..7486:+	1.41	0.00	0.95	2434.39
asi-miR-980-3p	UAGCUGCCUAGUGAAGGGC	19	KE524190.1:422320..422338:+	1.25	0.00	2.54	10.13
asi-miR-13-5p	UCGUAAAAAUGGUUGUGCUGUG	22	KE524905.1:90055..90038:+	0.63	0.00	0.63	0.00
asi-miR-iab-4-3p	CGGUAUACCUUCAGUAUACGUAAC	24	KE525233.1:295549..295572:−	0.63	0.00	0.00	0.00
asi-miR-2491-3p	CAACAACAGCAGCAGCAA	18	KL646034.1:671..688:+	0.00	0.00	0.16	0.00

### miRNA expression pattern during *An. sinensis* development

**Fig. 3:**
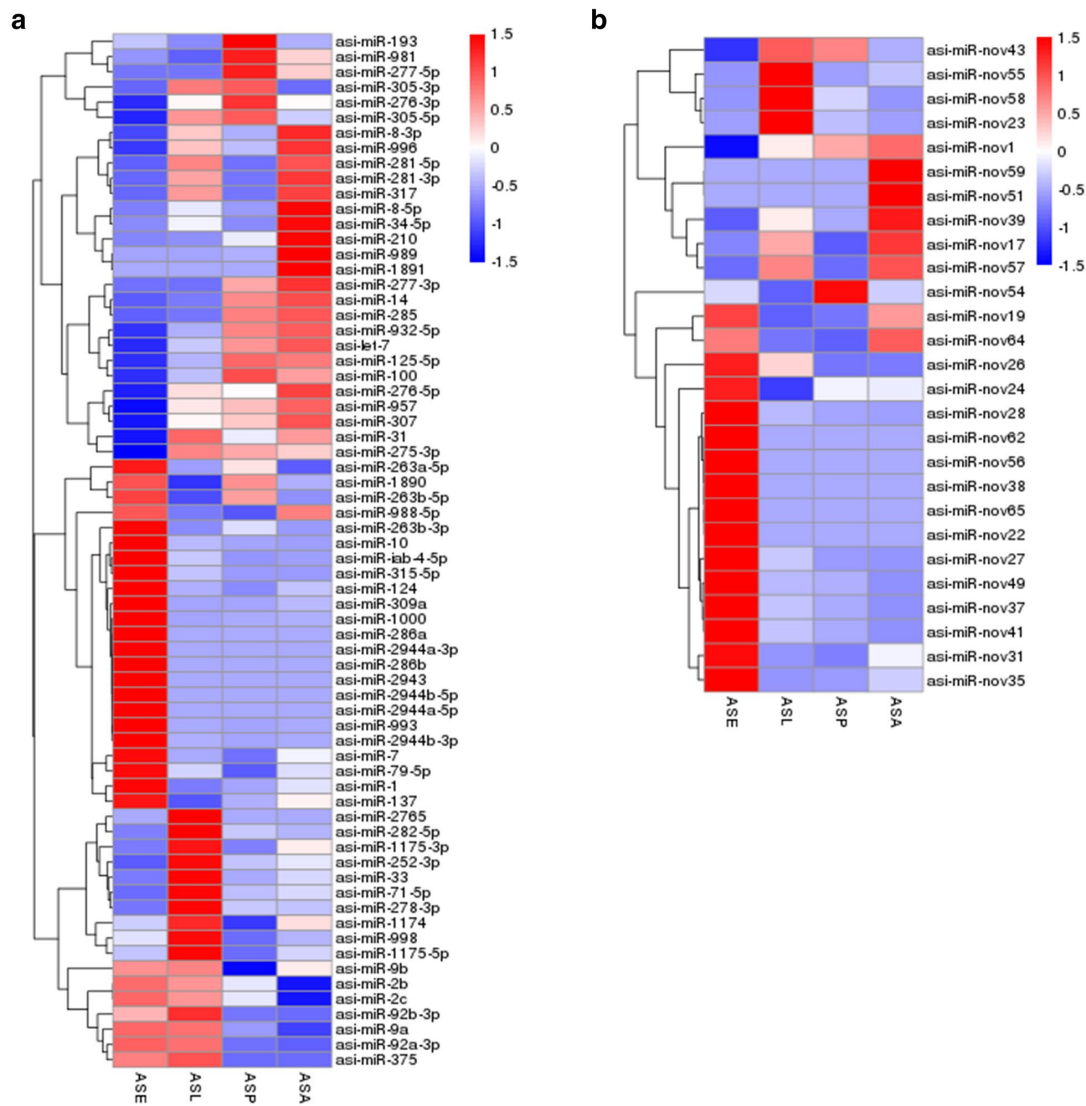
Heatmaps clustering of miRNAs expressed in the four life stage libraries of *An. sinensis*. The clustering was performed on all known (**a**) and novel (**b**) miRNAs based on TPM (tags per million) and the four life stage samples. Each row represents one miRNA and each column represents a stage. The color scale (shown on the left) illustrates the number of the reads of a miRNA across the life stages

The expression patterns of miRNAs in different life stages were examined based on the number of reads revised. The result showed that the expression profiles of miRNAs varied from highly specific to ubiquitous during the four stages. One-hundred and seventeen miRNAs were ubiquitously expressed in all developmental stages, while some miRNAs showed stage-specific patterns (Fig. 3). Among the known miRNAs, asi-miR-276-3p, asi-miR-1, asi-miR-100, asi-miR-8-3p, asi-miR-999, and asi-miR-9a showed constant high expression throughout four development stages. asi-miR-276-3p was the most frequently expressed miRNA in the larvae, the pupae, and the adult females’ library (TPM > 3.17 × 10^5^), whereas asi-miR-1 was the dominant miRNA in the eggs’ library (TPM > 1.87 × 10^5^). Nevertheless, asi-miR-219 had the lowest expression level in all developmental stages. asi-miR-2943 was specifically expressed in the embryo stage, and asi-miR-1891 could not be detected in the larval stage. The highly expressed novel miRNA in the eggs and pupae libraries was asi-miR-nov54 (TPM = 0.45 × 10^3^ and TPM = 1.50 × 10^3^), while in the larvae library and adult females’ library, it was asi-miR-nov55 (TPM = 0.91 × 10^3^) and asi-miR-nov1 (TPM = 0.42 × 10^3^), respectively. However, the expression level of novel miRNAs was generally low throughout all developmental stages when compared with known miRNAs.

### MicroRNA regulation from egg to adult during *An. sinensis* development

**Fig. 4:**
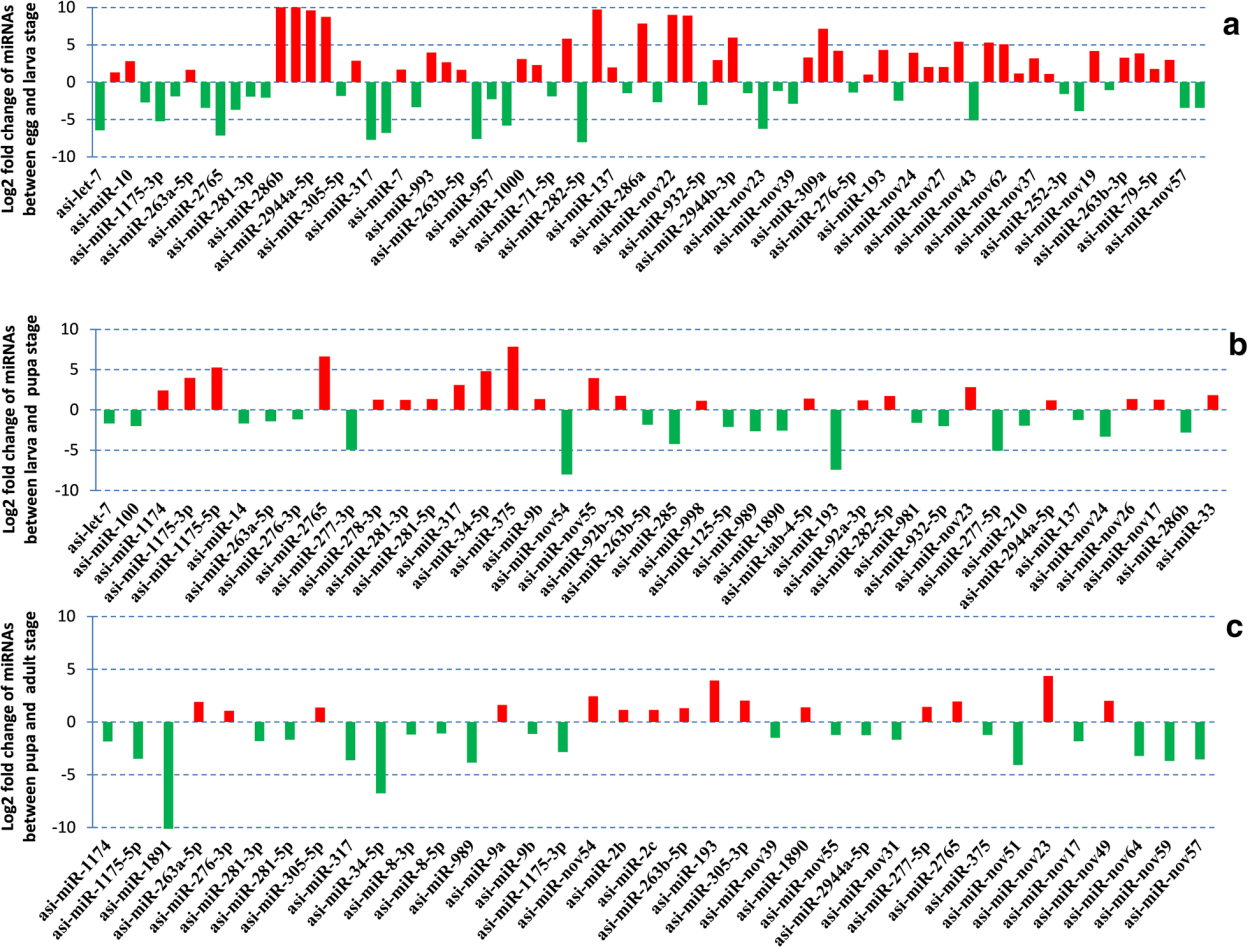
Fold-change of miRNAs during the development of *An. sinensis*. **a** Log2 fold change of miRNAs between embryo and larval stage, **b** Log2 fold change of miRNAs between larva and pupal stage, **c** Log2 fold change of miRNAs between pupal and adult stage. X axis represents miRNAs while Y axis represents fold-change of miRNAs

Hierarchical heat map revealed different expression patterns of the miRNAs between the two consecutive developmental stages (Fig. 3). Based on q-value and log fold change, 33 significantly down-regulated miRNAs (26 for known and 7 for novel miRNAs) and 41 significantly up-regulated miRNAs (25 for known and 16 for novel) were identified from the embyro to the larval stage. 20 down-regulated miRNAs (18 for known and 2 for novel miRNAs) and 22 up-regulated miRNAs (18 for known and 4 for novel) were observed form the larval to the pupal stage. 22 significantly down-regulated miRNAs (14 for known and 8 for novel miRNAs) and 15 significantly up-regulated miRNAs (12 for known and 3 for novel) were expressed form the pupal to adult stage (Fig. 4). It is noteworthy that the total number of dysregulated miRNAs decreased from the embryo to the adult stage. We also identified two known miRNAs (asi-miR-2943 and asi-miR-286b) to be significantly over-expressed (>tenfold) form the embryo to the larval stage and one known miRNA (asi-miR-1891) to be significantly under-expressed form the pupal to the adult stage (>tenfold) (Fig. 4). Sixteen miRNAs were differentially expressed in all developmental stages of the mosquito. Based on the analysis of these regulated miRNAs between the two consecutive developmental stages, five miRNAs (asi-miR-1890, asi-miR-193, asi-miR-263a-5p, asi-miR-263b-5p and asi-miR-nov54) were down-regulated in the larval stage whereas get up-regulated in the pupal stage but down-regulated in the adult stage again. Seven miRNAs (asi-miR-1175-3p, asi-miR-1175-5p, asi-miR-281-3p, asi-miR-281-5p, asi-miR-317, asi-miR-34-5p and asi-miR-nov55) were up-regulated in the larval stage whereas get down-regulated in the pupal but up-regulated in the adult stage again. asi-miR-nov23 and asi-miR-2765 were up-regulated in the larval stage whereas get down-regulated in both pupal and adult stage. asi-miR-2944a-5p was down-regulated in the larval and the pupal stages but up-regulated in the adult stage. asi-miR-989 was down-regulated in larval and pupal stages but up-regulated in the adult stages.

### Validation of differentially expressed miRNA

**Fig. 5:**
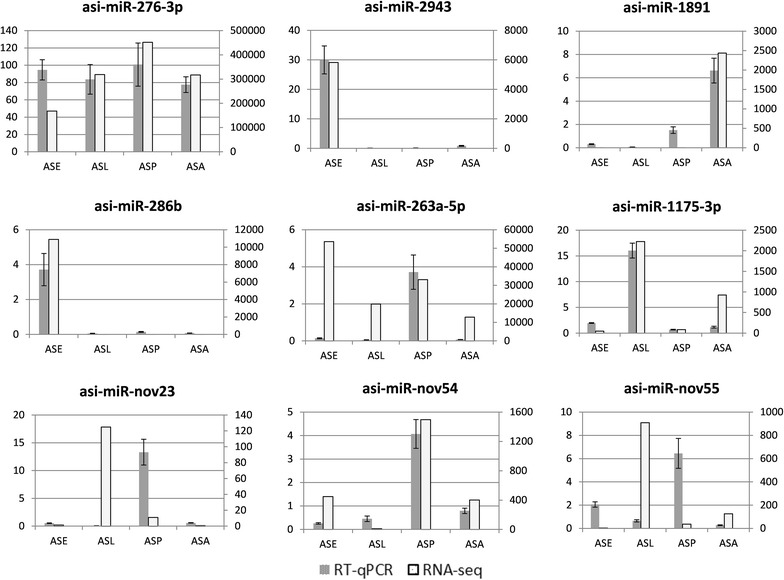
Validation of the differentially expressed miRNAs by Quantitative real-time PCR. The transcript levels of six miRNAs at different stages were calculated relative to the amount of *U6* small nuclear after normalization. The real time PCR data with bars represent a mean ± SD from three independent experiments

To validate the small RNA sequencing results, we performed quantitative real-time PCR analysis by selecting nine miRNAs that showed dramatic changes in expression level at different developmental stages. The result (Fig. 5) showed that six miRNAs (asi-miR-276-3p, asi-miR-2943, asi-miR-1891, asi-miR-286b, asi-miR-1175-3p, and asi-miR-nov54) showed similar expression patterns as those revealed by our sequencing result. However, three miRNAs (asi-miR-263a-5p, asi-miR-nov23, and asi-miR-nov55) expression levels detected by qRT-PCR were inconsistent with that of our sequencing results due to some unknown reasons.

### miRNA target gene prediction, GO enrichment, and KEGG pathway analysis

To understand the role of dysregulated miRNAs in physiological functions and biologic processes during development in *An. sinensis*, miRNA target gene prediction was performed to identify the putative target genes based on miRNA/mRNA complementarity. Multiple/different miRNA target prediction algorithms were applied to minimize the number of putative or false positive target genes. The target gene candidates of the differentially expressed miRNAs between two consecutive developmental stages of *An. sinensis* were predicted. A total of 7198 annotated mRNA transcripts were predicted as target gene candidates for 95 differentially expressed miRNAs (Additional file 3: Table S3). Among them, 5296 and 1902 target gene candidates were identified for 68 known and 27 novel miRNAs, respectively.

**Fig. 6:**
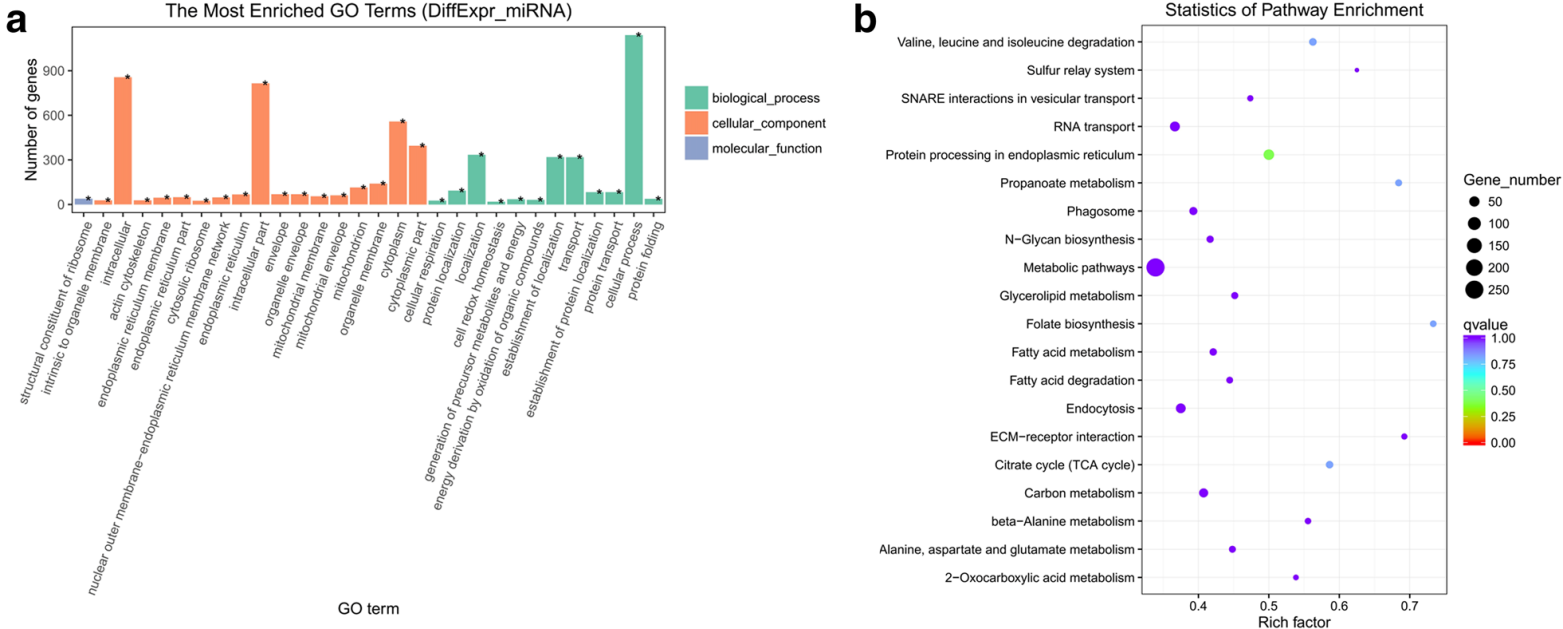
Gene ontology annotation by Goseq and KEGG pathway analysis by KOBAS for target genes of differentially expressed miRNAs. **a** Shows partial GO enrichment for the predicted target genes in ontologies of biological processes, cellular component, and molecular function. **b** Shows the enriched pathways for the predicted target genes during different stages of mosquito development

GO term annotation and KEGG Pathway analysis were used to further understand biological functions and signaling pathways that could potentially be regulated by these putative target genes. We found that the putative target genes of the differentially expressed miRNAs between two consecutive developmental stages of *An. sinensis* were involved in a broad range of biological functions and signaling pathways (Fig. 6). Detailed classifications about the biological process, cellular component, and molecular function of the miRNA targets were shown in Fig. 6a. By GO annotation, targets of miRNAs regulated from the embryo to the larval stage in the mosquito were enriched in intracellular, cytoplasm and cellular process. Targets of miRNAs regulated from larva to pupal stage in the mosquito were enriched in membrane part, membrane, cytoplasm and organic substance transport. Targets of miRNAs regulated from the pupa to the adult stage in the mosquito were enriched in structural molecule activity, membrane part and transport. Similarly, by KEGG pathway analysis, we found that 119 enriched pathways were identified in this study. The most enriched pathway included protein processing in endoplasmic reticulum, propanoate metabolism, folate biosynthesis, Citrate cycle (TCA cycle) and valine, leucine and isoleucine degradation (Fig. 6b).

### miRNA-gene network analyses identified key miRNAs and developmental genes

**Fig. 7:**
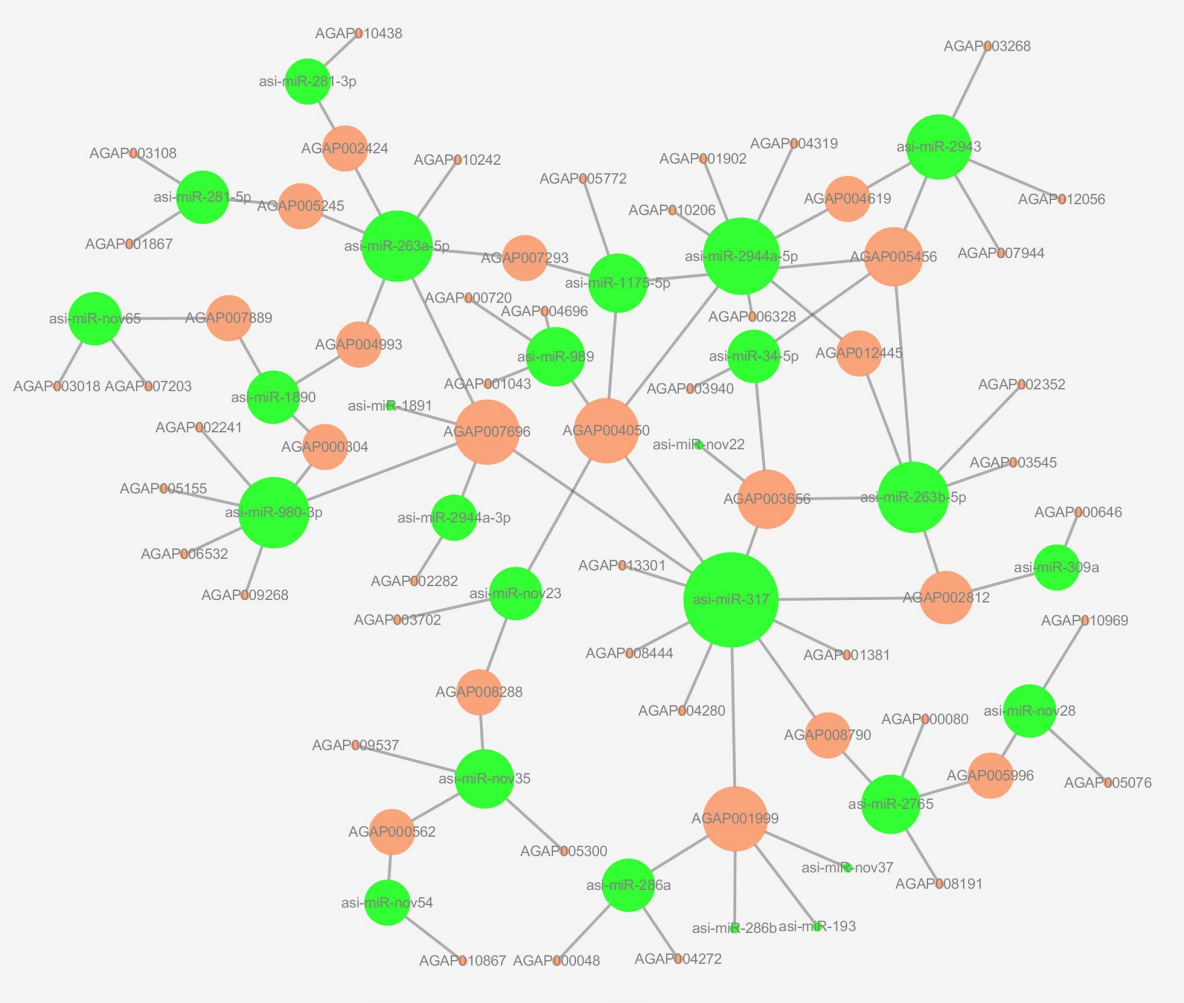
miRNA-mRNA interaction network predicted from 32 key miRNAs and 104 developmental genes. miRNAs are in green while mRNAs are in pink

Through analysis, 16 commonly dysregulated miRNAs identified from the miRNA expression files, 15 exclusively expressed miRNAs in certain stages (TPM > 10), and three the most significantly over/under-expressed miRNAs (>tenfold) were defined as the key miRNAs in *An. sinensis* development. A total of 546 developmental genes were obtained based on gene annotation in VectorBase and intensive literature surveys. After removing duplicates and invalid target genes, miRNA-gene network was built based on 32 key miRNAs and 104 developmental genes (Additional file 4: Table S4). We developed a network to demonstrate the overlapping miRNA targets for miRNAs since miRNAs usually have many mRNA targets. The number of targets for asi-miR-317, asi-miR-2944a-5p, asi-miR-263b-5p, asi-miR-263a-5p, and asi-miR-2943 was 10, 7, 6, 6 and 5, respectively (Fig. 7). This network also indicated that several mRNAs were predicted to be targets of one miRNAs. For example, AGAP001999, AGAP007696, AGAP005245, and AGAP004050 were targeted by two to five miRNAs, respectively (Fig. 7). As expected, the overall pattern suggests a positive correlation between the miRNA expression and developmental genes, such as AGAP000048 (*APC*), AGAP005449 (*Cbl*), AGAP001867 (*hep*), which encoding important components of major developmental signaling pathways.

## Discussion

In the present study, the miRNA expression profile of *An. sinensis* in four different life stages was analyzed by NGS technology, and approximately 100 million high-quality reads were obtained. The mapping rates of high-quality reads to the *An. sinensis* genome were between 77.61 and 85.75%. This might be subject to incomplete reference genome. The size distribution of small RNA sequence reads showed peaks between 20 AND 30 nucleotides comparing to the average miRNA length. Annotation of the sRNAs in the four libraries indicated that the most abundant class of sRNAs in the eggs, larvae, pupae and adult females’ libraries was miRNAs, rRNAs (larvae and pupae) and tRNAs respectively. This result is consistent with previous research in *Anopheles funestus* [37], indicating that protein synthesis during development is of great importance.

In total, 99 known miRNAs and 65 novel miRNAs expressed in the four life stages of the *An. sinensis* were identified. As expected, most of the known miRNAs identified in *An. sinensis* were highly conserved across diverse mosquito species, suggesting that the conserved miRNAs may involve in many critically fundamental regulatory pathways. Novel miRNAs found in this study are not conserved in the *Anopheles* genus, and we cannot find homologs in other insect species by searching the miRBase, suggesting that these might be species-specific miRNAs. However, the predicted novel miRNAs exhibited much lower expression levels when compared with conserved miRNAs, which is also consistent with previous reports [9, 37]. Previously, Dritsou and his colleagues [6] computationally predicted 115 miRNAs (after removing 33 duplicates and 11 putatively new miRNA, 71 left) in *An. sinensis* from the genome level. We compared our miRNA dataset with theirs and found that 33 miRNAs overlap with our predictions. Among these miRNAs, 19 miRNAs could be found in *Ae. aegypti*, *An. gambiae*, and *Cu. quinquefasciatus.* Six miRNAs (miR-193, miR-2765, miR-277-5p miR-2b, miR-315-5p and miR-9b) were only found in *Ae. aegypti*. In contrast, we validate more miRNAs existence and detect their expression pattern at different developmental stages.

To obtain insight into possible stage-specific miRNAs and their roles in *An. sinensis*, we investigated the expression profile of the miRNAs identified in the four life stage libraries. One-hundred and seventeen miRNAs were ubiquitously expressed in all developmental stages. The top five most highly expressed miRNA across four developmental stages were asi-miR-276-3p, asi-miR-1, asi-miR-100, asi-miR-9a and miR-8, which is basically in line with previous report in other mosquito species [15, 38]. The most highly expressed miRNA in female *An. sinensis* was asi-miR-276-3p. In contrast, Skalsky et al. [15] reported that miR-184 was the most highly expressed miRNA in C7/10 *Ae. albopictus* cells and blood-fed female *Cx. quinquefasciatus* mosquitoes [15]. *afu*-*miR*-*8* was the dominant miRNA in the unfed adult *An. funestus* females [37]. In addition, miR-1, miR-184, and miR-263 ranked among the most highly expressed miRNAs in both *Ae. aegypti* and *An. stephensi* [27]. aga-bantam, aga-miR-263a, aga-miR-8, aga-miR-10, aga-miR-184 and aga-miR-281 were the most abundantly expressed miRNAs in *An. gambiae* [38]. Thus, despite the differences in expression level, most of these reported abundant miRNAs in different mosquito lineages are still among the top 10 most highly expressed miRNA in *An. sinensis*.

Comparisons between egg and larva, larva and pupa, and pupa and unfed adult female are shown in Figs. 3 and 4. A majority of miRNAs were differentially expressed in the four stages. For example, 41 miRNAs were up-regulated and another 33 down-regulated between the embryo and larva stage. Especially, asi-miR-2943 and asi-miR-286b were up-regulated more than tenfold change. While between the larva and the pupa, 22 miRNAs were up-regulated and 20 other miRNAs were down-regulated. We also identified 15 significantly up-regulated miRNAs and 22 significantly down-regulated miRNAs from the pupal to the adult stage. And asi-miR-1891 was significantly down-regulated in this process. Dramatic alternation of miRNA expression during these three stages signifies that these miRNAs may be involved in the regulation of metamorphosis during development. What’s more, asi-miR-2943 was exclusively expressed in the embryo stage, which is also highly expressed in the embryos in both *Ae. aegypti*, *An. stephensi* [39] and *An. anthropophagus* [17], indicating its important roles in embryogenesis across many mosquito species. However, miR-2943 was present at low levels in both *Cx. quinquefasciatus* and *Ae. albopictus* C7/10 cells [15]. Similarly, miR-1891 was most abundantly expressed in *Ae. aegypti* and *An. stephensi* pupal stage [39], however, asi-miR-276-3p was the highest expressed miRNAs in *An. sinensis* pupae, although asi-miR-1891 also exhibited a high expression. These results indicate that, on the one hand, miRNA profiles of mosquito lineage may differ from each other, one the other hand, specifically expressed miRNAs or regulatory miRNAs during developmental stages may also be different. In addition, sample preparation, detection methods and sensitivity may also lead to inconsistency between different studies.

Moreover, we observed that the expression level of miRNAs did not correlate with the fold change. Some miRNAs showed higher expression but with low fold changes. For instance, asi-miR-276-3p, asi-miR-1, asi-miR-100 and asi-miR-7 were among the top 10 most highly expressed miRNAs in all developmental stages but showed low log2-fold changes. However, asi-miR-193 and asi-miR-nov54 were low expressed miRNAs in *An. sinensis* developmental stages but showed high log2-fold changes. Additionally, asi-miR-309a, asi-miR-2943, asi-miR-315-3p and asi-miR-iab-4-3p could not be detected in the pupae and larvae libraries, asi-miR-275-5p and asi-miR-2491-3p was not identified in the pupae and adult females’ libraries, and asi-miR-980-3p was not found from the pupae library. These results indicate that the expressions of some miRNAs are probably stage specific. We assumed that stage-specific miRNAs might be involved in regulation of embryogenesis, organ differentiation, growth, and reproduction during a specific developmental stage. Investigation on stage-specific miRNAs may detail understanding of mosquito biology and could provide mosquito-specific targets for disease control. However, the function of majority of the miRNAs remains to be determined.

To confirm our sequencing results, we performed quantitative real-time PCR analysis using the RNA samples used for small RNA NGS. Thus, nine miRNAs that showed dramatic changes in expression level at different developmental stages were selected for validation. All nine miRNAs (asi-miR-276-3p, asi-miR-2943, asi-miR-1891, asi-miR-286b, asi-miR-1175-3p, asi-miR-263a-5p, asi-miR-nov23, asi-miR-nov54, and asi-miR-nov55) could be detected in the sample. Six miRNAs showed similar expression patterns as those revealed by our sequencing analysis, which indicated a low false discovery rate of our sequencing data and supported the validity of the profiling. However, the expression levels of asi-miR-263a-5p, asi-miR-nov23, and asi-miR-nov55 detected by qRT-PCR were inconsistent with that of our sequencing results. We inferred that this might be caused by the experimental conditions, such as base bias, amplification efficiency, and analytical error in both small RNA NGS and the qRT-PCR, but some other unknown reasons cannot be excluded.

Mosquitoes grow through four phases of metamorphosis: egg, larva, pupa and adult. Knowing the different stages of the mosquito’s life will help us to prevent mosquitoes and disease transmission. To further understand the role of miRNAs in *An. sinensis*, miRNA target gene prediction was performed based on miRNA/mRNA interactions to provide molecular insight into their function by using two computational methods. A total of 7198 annotated mRNA transcripts were predicted as putative target genes for 95 differentially expressed miRNAs. The enriched biological functions and signaling pathways with significant role at a specific stage of development were predicted by GO term annotation and KEGG Pathway analysis. Metamorphosis from embryo to larval and from pupal to adult stage involves biological functions such as cellular component, membrane, cytoplasm and organic substance transport. In addition, enriched pathways involved in Protein processing in endoplasmic reticulum, Propanoate metabolism, Folate biosynthesis, Citrate cycle (TCA cycle) and Valine, leucine and isoleucine degradation were found. These biological functions and pathways might be important for degeneration and formation of tissue/organ, which happens during immature stages to complete its transition from egg to adult.

Network of miRNA-mRNA interactions was predicted by using results from miRNA analysis and mRNA retrieved from developmental genes of *An. gambiae* and then visualized by Cytoscape. Further analyses indicated that a number of miRNAs (asi-miR-317, asi-miR-2944a-5p, asi-miR-263b-5p, asi-miR-263a-5p, and asi-miR-2943) target a cluster of mRNA and vice versa. The candidate targets mentioned here have been reported to be key components in developmental pathways [31]. For example, AGAP002352, one of the targets of asi-miR-263b-5p, is an annotated apoptosis-related gene (*p53*) in *Ae. aegypti* and many other mosquitoes. *p53* plays an important role in programmed cell death in relation to development [40]. Also, AGAP003108 (*htl*), AGAP001867 (*hep*) and AGAP005245 (*lola*) were the common target of asi-miR-281-5p. *htl* is one of two FGF (Fibroblast Growth Factor) receptors, and FGF signaling regulates a variety of biological processes during development, such as cell differentiation and cell proliferation [41]. *hep* is one component of Ras signaling pathways which also could regulate many developmental processes [42]. *lola*, which is called axon guidance gene that regulate wiring of the olfactory system in *D. melanogaster* [43], have been reported to have many orthologs in mosquito [31]. In addition, knock-in and knock-down of specifically and temporally expressed miRNAs in *Ae. albopictus* by microinjection have improved our understanding of regulated miRNAs in mosquito development [16]. Intervention on aal-miR-286b led to a profound decrease in the hatching rate of embryos. In addition, eclosion rate of larvae in aal-miR-2942 knocked-down group showed a significant decrease compared to control group. A marked reduction in longevity and fecundity was also observed in the miR-1891 inhibitor group. These research findings have established the exact functional roles these developmental genes and miRNAs in regulating the stage-specific development. However, additional extensive experimental analyses are required to confirm the interactions of miRNA-gene regulatory role in many phenotypic changes during developmental stages in further ongoing research work.

Although miRNAs are reported to play a role in development in *C. elegans*, fruit flies and other insects, and significant stage-specific expression was observed for a number of miRNAs in various mosquito species. However, the role of miRNAs in metamorphosis and development is far less understood and remains an enigma. For some specific or novel miRNAs, their functional roles still need to be confirmed by biological experiments. Furthermore, only very limited specific target genes have been identified for small part of miRNAs. It is important to note that the result in this work is a key step towards improving our understanding of the complexity and regulation mode of miRNAs in *An. sinensis* embryogenesis and metamorphosis. Further analysis of stage-specific miRNA expression and functions would be helpful to decipher the complex genetic network that controls mosquito at a crucial stage.

*Anopheles sinensis* is the most common and wide-distributed malaria vector exhibiting a variety of ecological behaviors and feeding habits throughout the China. It is also of great medical and veterinary importance. Thus, identification of key miRNAs required for gene regulation throughout the life cycle will not only help to a better understanding of its developmental biology but also may help to conceive novel approaches to control this vector. However, the function and molecular mechanism of these miRNAs are still need to be further investigated.

## Conclusions

In conclusion, this study provides a comprehensive account of miRNA profile and its dysregulated expression patterns across four different stages of *An. sinensis* development using the high throughput sequencing. Several miRNAs show stage-specific expression pattern in particular developmental stages. We also investigate targets of these dysregulated miRNAs and their related functions in important life activities in order to provide a better understanding of mosquito development. In turn, such information would provide the basis to conceive novel approaches to control this vector in future.

## Additional files

**Additional file 1: Table S1.** Primers used in qRT-PCR validation.

**Additional file 2: Table S2.** Novel miRNAs identified in four life stages libraries of *An. sinensis*.

**Additional file 3: Table S3.** Target gene candidates for known and novel miRNAs in *An. sinensis*.

**Additional file 4: Table S4.** Developmental target gene candidates for 32 key miRNAs during development in *An. sinensis*.

## Abbreviations

*An. sinensis*: *Anopheles sinensis*
*P. vivax*: *Plasmodium vivax*
*An. gambiae*: *Anopheles gambiae*
*An. stephensi*: *Anopheles stephensi*
*Ae. aegypti*: *Aedes aegypti*
*Cu. fatigans*: *Culex fatigans*
*Ae. albopictus*: *Aedes albopictus*
*An. anthropophagus*: *Anopheles anthropophagus*
miRNA: microRNA
Pre-miRNA: precursor microRNA
ASE: *An. sinensis* egg
ASL: *An. sinensis* larvae
ASP: *An. sinensis* pupa
ASA: *An. sinensis* adult female
piRNA: Piwi-interacting RNA
nt: Nucleotide
GO: gene ontology
KEGG: kyoto encyclopedia of genes and genomes
UTR: untranslated region
NGS: next generation sequencing
TPM: tags per million
mRNA: messenger RNA
rRNA: ribosomal RNA
tRNA: transfer RNA
small ncRNAs: small non-coding RNAs
snoRNA: small nucleolar RNA
qRT-PCR: reverse transcription quantitative real-time polymerase chain reaction
